# Psychological Stress as a Risk Factor for Accelerated Cellular Aging and Cognitive Decline: The Involvement of Microglia-Neuron Crosstalk

**DOI:** 10.3389/fnmol.2021.749737

**Published:** 2021-11-04

**Authors:** Micaël Carrier, Eva Šimončičová, Marie-Kim St-Pierre, Chloe McKee, Marie-Ève Tremblay

**Affiliations:** ^1^Axe Neurosciences, Centre de Recherche du CHU de Québec, Université Laval, Québec City, QC, Canada; ^2^Division of Medical Sciences, University of Victoria, Victoria, BC, Canada; ^3^Department of Molecular Medicine, Université Laval, Québec City, QC, Canada; ^4^Department of Biology, University of Victoria, Victoria, BC, Canada; ^5^Neurology and Neurosurgery Department, McGill University, Montreal, QC, Canada; ^6^Department of Biochemistry and Molecular Biology, University of British Columbia, Vancouver, BC, Canada

**Keywords:** microglia, neuron, synapse, cognitive aging, oxidative stress, chronic psychological stress, major depressive disorder

## Abstract

The relationship between the central nervous system (CNS) and microglia is lifelong. Microglia originate in the embryonic yolk sac during development and populate the CNS before the blood-brain barrier forms. In the CNS, they constitute a self-renewing population. Although they represent up to 10% of all brain cells, we are only beginning to understand how much brain homeostasis relies on their physiological functions. Often compared to a double-edged sword, microglia hold the potential to exert neuroprotective roles that can also exacerbate neurodegeneration once compromised. Microglia can promote synaptic growth in addition to eliminating synapses that are less active. Synaptic loss, which is considered one of the best pathological correlates of cognitive decline, is a distinctive feature of major depressive disorder (MDD) and cognitive aging. Long-term psychological stress accelerates cellular aging and predisposes to various diseases, including MDD, and cognitive decline. Among the underlying mechanisms, stress-induced neuroinflammation alters microglial interactions with the surrounding parenchymal cells and exacerbates oxidative burden and cellular damage, hence inducing changes in microglia and neurons typical of cognitive aging. Focusing on microglial interactions with neurons and their synapses, this review discusses the disrupted communication between these cells, notably involving fractalkine signaling and the triggering receptor expressed on myeloid cells (TREM). Overall, chronic stress emerges as a key player in cellular aging by altering the microglial sensome, notably via fractalkine signaling deficiency. To study cellular aging, novel positron emission tomography radiotracers for TREM and the purinergic family of receptors show interest for human study.

## Introduction

Since their discovery, microglia have been described as the resident macrophages of the central nervous system (CNS). They migrate from the yolk sac to the brain around embryonic day 9.5 in mice (Ginhoux et al., 2010) and can be observed for the first time in the developing human brain around the 4.5–5th week of gestation (Andjelkovic et al., 1998; Monier et al., 2006; Verney et al., 2010). Following their migration and colonization, microglia remain distributed unevenly between the gray and white matters and across individual brain regions (Lawson et al., 1990; Mittelbronn et al., 2001). Indeed, Lawson et al. (1990) described the distribution of microglia in adult mice and found that these immune cells ranged from 5 to 12% of all brain cells. In humans, microglial distribution, which was discovered to be greater in the white matter, varied from 0.3 to 16.9% of all brain cells depending on the region (Mittelbronn et al., 2001). Microglial numbers are then maintained through local self-renewal in physiological conditions, a phenomenon conserved across species (Lawson et al., 1992; Askew et al., 2017; Füger et al., 2017; Réu et al., 2017; Tay et al., 2017).

Microglia are categorized most notably by their morphological state (e.g., surveillant, bushy or hyper-ramified, ameboid, dystrophic) and molecular signature (Martinez and Gordon, 2014; Ransohoff, 2016; Stratoulias et al., 2019). While previously termed as “quiescent” or “resting,” surveillant microglia are quite dynamic; their ramified processes retract and extend, constantly surveying the parenchyma for environmental cues (Davalos et al., 2005; Nimmerjahn et al., 2005). This allows them to interact with numerous cellular elements including astrocytic and neuronal cell bodies, synapses, but also with the basement membrane of the brain vasculature (Tremblay et al., 2010; Bisht et al., 2016; Stogsdill and Eroglu, 2017; Joost et al., 2019; Matejuk and Ransohoff, 2020; Vainchtein and Molofsky, 2020). Microglia contacting neuronal cell bodies are known as “satellite” microglia (Wogram et al., 2016; Stratoulias et al., 2019), a position recently associated with the regulation of neuronal activity (Cserép et al., 2020). Another interesting aspect of microglia is their ability to quickly alter morphologically to appropriately respond to the functional needs of the brain. Once microglia detect CNS insults through local stimuli, they undergo drastic morphological transformations. Their morphology can range from ameboid to hyper-ramified states (Davalos et al., 2005; Nimmerjahn et al., 2005; Wake et al., 2009), tightly following changes in their transcriptome and proteome (Reynolds et al., 2008; Dungrawala et al., 2010; Zhurinsky et al., 2010; Marguerat and Bähler, 2012; Rangaraju et al., 2018; Bell et al., 2019; Miedema et al., 2020; Rayaprolu et al., 2020). This microglial remodeling is observed in conditions of psychological stress, where among various brain regions that include the prefrontal cortex (PFC) and hippocampus, microglia were found to dynamically transform their morphology, gene and protein expression, as well as function (Bordeleau et al., 2020; Picard et al., 2021). These microglial changes to stress become further exacerbated with aging (Tay et al., 2017). Psychological stress is well-known to accelerate cellular aging (Yegorov et al., 2020), hence potentially predisposing to various neuropsychiatric disorders and neurodegenerative diseases across the lifespan (Musazzi and Marrocco, 2016; Justice, 2018; Desmarais et al., 2020; Smith and Pollak, 2020).

Microglia entertain a particular relationship with neurons. Developmental studies taught us that microglia notably influence neurogenesis by controlling the maturation, density and migration of neuronal progenitors during the first weeks of life (Paolicelli and Gross, 2011; Bordeleau et al., 2019). Acting specifically on neurons, microglia can influence their axonal growth (Pont-Lezica et al., 2014) and ability to form synapses by inducing the genesis of filopodia at dendritic elements (Miyamoto et al., 2016). Microglia are key players in synaptic plasticity, both structural and functional, which allows them to modify the neuronal circuitry via synaptic pruning, synaptic stripping, the secretion of neurotrophic factors and regulation of synaptic activity (Ji et al., 2013a,b; Sipe et al., 2016; Zhou et al., 2019), phenomena confirmed in the olfactory bulb, hippocampus, as well as cerebral cortex in mice (Tremblay and Majewska, 2011; Kettenmann et al., 2013; Morris et al., 2013; Schafer et al., 2013; Milior et al., 2016; Miyamoto et al., 2016). Microglia can modulate learning and memory, including auditory-cued fear conditioning and novel object recognition. This was for instance shown in mice depleted of microglia, which displayed altered performance in these tasks (Parkhurst et al., 2013; Torres et al., 2016). During early postnatal development, microglia are also able to shape the visual cortex of mice in an experience-dependent manner (Tremblay et al., 2010). By sensing synaptic activity, microglia remove weaker signaling synapses in the visual thalamus (Schafer et al., 2013). This is an important concept, as the best correlate for cognitive decline is synaptic loss (Scheff et al., 2006; Jackson et al., 2019; Colom-Cadena et al., 2020). Reduced synaptic density in the PFC is observed upon chronic psychological stress (Csabai et al., 2018), although some studies also show region-specific synaptic growth, for instance in the amygdala, in association with an emotional memory component (Picard et al., 2021). These various roles of microglia in the healthy brain were additionally shown to differ between sexes (Bordeleau et al., 2019).

In this review, we describe recent discoveries on the bidirectional communication between microglia, neurons, and their synapses, as well as discuss how their interactions are altered upon psychological stress and in major depressive disorder (MDD), leading to accelerated brain and cognitive aging. Studies on these emerging mechanisms in humans by positron emission tomography (PET) imaging are also briefly mentioned because of their potential to investigate MDD. Previous investigations using PET imaging have shown decreased metabolites in the limbic lobe and basal ganglia of MDD patients (Su et al., 2014). Further investigation tracking microglia-neuron receptor/ligand communication could offer monitoring opportunity for disease progression and treatment response in MDD patients.

## Microglia-Neuron Communication and Interaction in Health, Stress, and Aging

The communication between microglia and neurons is bidirectional (Tremblay, 2011; Eyo and Wu, 2013; Szepesi et al., 2018), essential for homeostatic function and implicated in neurodegenerative disorders (Sheridan and Murphy, 2013). It is achieved via the complex microglial sensome constituting a myriad of various key receptors that constantly receive signals from surrounding neurons (Hickman et al., 2013) as well as their synapses (Roumier et al., 2004, 2008; Bessis et al., 2007; Béchade et al., 2013; Cantaut-Belarif et al., 2017; Tay et al., 2017; Comer et al., 2020). Several essential molecular mechanisms such as fractalkine signaling, the classical complement pathway, purinergic signaling, and triggering receptor expressed on myeloid cells 2 (TREM2) are discussed below. Diverse types of signaling can influence each other, acting as on and off systems (Biber et al., 2007).

### Fractalkine Signaling

A main mediator of this communication is signaling between the neuronal chemokine fractalkine (CX3CL1) and its unique receptor (CX3CR1) expressed on the surface of microglia (Harrison et al., 1998; Ransohoff and Perry, 2009; Paolicelli et al., 2014; Lauro et al., 2019). During development, neurons expressing CX3CL1 (Meucci et al., 1998) modulate microglial pruning of synapses and brain functional connectivity, thus participating to proper brain maturation and social behavior in mice (Zhan et al., 2014; Arnoux and Audinat, 2015; Gunner et al., 2019). In adult mice, loss of fractalkine signaling causes widespread deficits in glutamate release at hippocampal synapses, which are associated with defects of adult hippocampal neurogenesis as well as learning and memory (Maggi et al., 2009; Rogers et al., 2011; Paolicelli et al., 2014; Zhan et al., 2014; Basilico et al., 2019), while psychological stress is associated with lower CX3CL1-CX3CR1 signaling between neurons and microglia (Han et al., 2019b). Throughout life, fractalkine signaling plays a part in the stress response, as CX3CR1 knockout in mice delays or prevents the response to chronic stress (Hulshof et al., 2003; Wohleb et al., 2013; Milior et al., 2016; Rimmerman et al., 2017; Winkler et al., 2017). In aging, fractalkine signaling is important for the regulation of adult neurogenesis in the hippocampus, but not the olfactory bulb (Gemma et al., 2010; Reshef et al., 2017; Bolós et al., 2018). However, hippocampal fractalkine signaling decreases steadily over the course of aging in mice, where its consequences still remain unknown (Mecca et al., 2018). Moreover, young adult (2-month old) mice with knockout for CX3CR1 were demonstrated to have a microglial transcriptome resembling that of aged mice based on its expression of inflammatory genes, suggesting a protective role of fractalkine signaling in aging (Gyoneva et al., 2019).

### Classical Complement Pathway

Complement signaling is an essential part of the innate immune system bearing the role of opsonization, the secretion of molecules enhancing phagocytosis, contributing to overall pathogen removal (Veerhuis et al., 2011). Growing evidence places the complement protein 3 (C3) as essential for proper brain development (Lee et al., 2019), considering that C3 guides microglial synaptic pruning (Schafer et al., 2012; Luchena et al., 2018). In aging rhesus monkeys, the expression of C1q protein, an upstream complex of C3, is increased within synaptic elements of the PFC, signaling to microglia which synapses need to be pruned (Datta et al., 2020). Evidence for an implication of the complement pathway in stress-related disorders shows increased C3 expression in the PFC of mouse models of stress-induced depressive-like disorder (Crider et al., 2018). Distress paradigm in mice has been associated with hyper-ramified microglia and a loss of dendritic spines, paralleled by an increased expression of C1q component in the medial PFC and hippocampus (Smith et al., 2019). These observations are in agreement with an initially beneficial immune response, as shown by other mouse studies, where chronic stress was found to initially increase microglial survival through colony stimulating factor 1 (CSF1) signaling, which additionally promoted neuronal remodeling via the complement pathway (Kreisel et al., 2014; Wohleb et al., 2018; Horchar and Wohleb, 2019). Prolonged stress reduced microglial numbers, hence preventing effective neurogenesis, but this deficit was rescued by stimulating CSF1 signaling (Kreisel et al., 2014).

### Purinergic Signaling

Purinergic receptors, many of which are highly expressed by microglia, can be separated into two families, i.e., sensors of adenosine (P1) counting A1, A2A and A3 or nucleotides (P2), which include the receptors P2Y12, P2Y6, P2Y4, P2X4, P2X7, and pannexin 1 (Burnstock, 2013; Illes et al., 2020; Sanchez-Arias et al., 2021). During development and injury, P2Y12 is a key adenosine triphosphate (ATP)-sensitive receptor responsible for determining microglial process motility, an important aspect involved in phagocytosis of both cellular debris or synaptic elements, notably in the mouse visual system (Haynes et al., 2006; Eyo et al., 2014; Sipe et al., 2016). This process was also characterized at the microglial filopodia level, showing that these structures perform a nanoscale surveillance of the mouse brain in homeostasis (Bernier et al., 2019). As microglia sense ATP to put themselves in motion, they also communicate with neurons via the secretion of adenosine binding to adenosine A1 receptor (A1R), which is able to act as negative feedback to suppress neuronal activity by limiting synaptic transmission (Badimon et al., 2020). During prolonged stress exposure, P2X7 receptor is up-regulated in hippocampal and PFC microglia, initiating an inflammatory response via the NOD-, LRR- and pyrin domain-containing protein 3 (NLRP3) inflammasome (Franklin et al., 2018; Dias et al., 2021). Assembly of the NLRP3 inflammasome via the P2X7 receptor was shown in the rat hippocampus to take place after 3 weeks of chronic stress (Yue et al., 2017). The implication of purinergic receptors in aging is still elusive, but cognitive decline-related disease conditions involving inflammation, such as Alzheimer’s disease (AD), show a consistent up-regulation of P2X7 in microglia (Woods et al., 2016; Francistiová et al., 2020; Pietrowski et al., 2021).

### Triggering Receptor Expressed on Myeloid Cells 2 Signaling

TREM2 is a cell surface receptor for putative ligands that include lipids, DNA and pro-inflammatory proteins such as apolipoprotein E and amyloid (β) oligomers, which activate downstream signaling pathways for cell survival, phagocytosis, metabolic fitness and cell motility (Ulland et al., 2017; Konishi and Kiyama, 2018). While TREM2 is a major pathology-induced immune signaling pathway (Deczkowska et al., 2020), it is also required for synaptic pruning during normal development (Filipello et al., 2018). Cell survival-regulating Wnt/β-catenin pathways that are up-regulated after TREM2 binding, notably by one of its ligands Transmembrane Protein 59 (TMEM59), bear great importance for microglia. Knockout of the receptor impairs microglial response in AD, leading to an exacerbated sensibility to stress-induced cell death (Zheng et al., 2017; Liu et al., 2020; McQuade et al., 2020), with reports highlighting the emergence of susceptibility only upon TREM2 haploinsufficiency (Sayed et al., 2018). Later in life, TREM2 sustains a key signaling pathway for microglia, allowing their clearance of myelin debris (Kober and Brett, 2017). This role in myelin recycling is also key in AD, as mutant microglia for TREM2 overly produce autophagic vesicles via a deficit in the mammalian target for rapamycin (mTOR) pathway, leading to AD pathologies in the 5XFAD mouse model (Ulland et al., 2017). Furthermore, aged TREM2 knockout mouse microglia show reduction of phagocytosis and sudden increase in cellular oxidative stress (Linnartz-Gerlach et al., 2019). Overall, TREM2 seems to be major signaling pathway throughout life and shows a great pathological risk when altered in aging.

### Tyro3, Axl, and Mer Receptors

Tyro3, Axl, and Mer (TAM) receptors are part of the cellular tyrosine kinase (TK) signaling pathways of the brain (Lemke, 2013). Microglial TAM receptors comprised of Axl and MerTK were shown to be required for their functions during normal physiological conditions as well as injury, specifically their process motility and phagocytosis of apoptotic newborn neurons (Fourgeaud et al., 2016). This is important as TAM receptors can promote hippocampal neurogenesis by preventing the secretion of inflammatory cytokines, as shown *in vitro* using a triple (Tyro3, Axl, MerTK) knockout model for these receptors with cultured microglia (Ji et al., 2013c). During aging, the Axl pathway is implicated in amyloid β clearance, hence preventing cellular toxicity in mice (Zhang et al., 2018) and with amyloid plaque-associated microglia expressing Axl and MerTK (Savage et al., 2015). However, the role of microglial TAM receptors during stress and aging remains elusive.

## Microglial Metabolic and Morphological Changes in Stress and Aging

**FIGURE 1:**
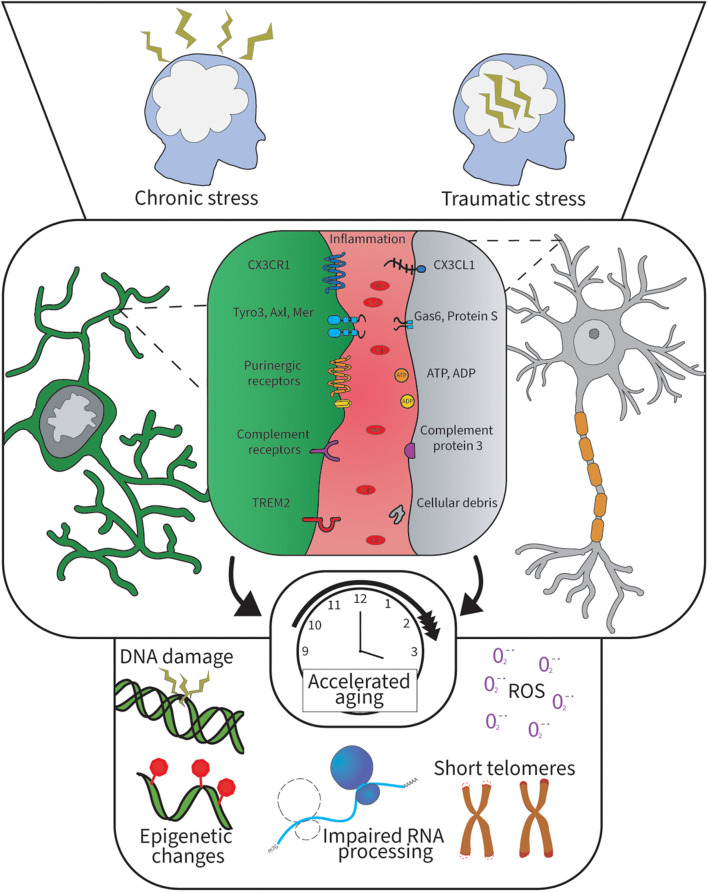
Psychological stress and inflammation alter microglia-neuron communication and lead to cellular aging. Psychological stress induces brain inflammation and oxidative stress resulting in cellular aging when prolonged. This hostile brain environment disrupts the microglia-neuron brain communication and leads to cognitive decline. The top panel illustrates two major types of stress lived by humans. The middle panel shows the different microglia-neuron signaling pathways in the brain and the key inflammatory factors identified in chronic stress. The bottom panel illustrates the different cellular aging processes happening when cells undergo cellular aging, which is often associated with inflammation and aging.

Immune dysregulation crucially impacts an individual’s ability to cope with internal and external challenges, such as trauma, infection, but also psychological stress, altering future response to stress outcomes in life and promoting cellular aging (Godbout et al., 2005; Buchanan et al., 2010; O’Donovan et al., 2012; Ritzel et al., 2019). A combination of several mechanisms including cellular senescence, deficits in energetic metabolism, release of inflammatory mediators, dysregulation of protein degradation pathways, activation of DNA repair mechanisms and gut dysbiosis, were suggested to be involved in cellular aging (Franceschi and Campisi, 2014; Franceschi et al., 2018). Upon exposure to immune challenges, stress or pathological aging, microglia are presumed to undergo a *priming* process (Püntener et al., 2012; Frank et al., 2018; Keane et al., 2021) leading to an elevated basal inflammatory activity [i.e., production of tumor necrosis factor alpha (TNFα), interleukin (IL)-1 beta (IL-1β), IL-6] (Sierra et al., 2007; Frank et al., 2010; Marschallinger et al., 2020), further increasing the overall inflammation levels in the brain (Franceschi and Campisi, 2014). Recent evidence showed that microglial priming can be explained by the trained immunity hypothesis, in which a challenge trains microglia to a next insult by promoting their secretion of intracellular metabolites and changing their epigenetic program (Haley et al., 2019; Neher and Cunningham, 2019). These findings are in line with increased gene expression within the interferon (IFN)-pathways observed in rodent models of aging (Grabert et al., 2016; Raj et al., 2017), and result in an inflammatory environment for microglia and neurons (Figure 1; Carnevale et al., 2007).

Microglial populations become more heterogeneous with aging as compared to the adult life stage (Olah et al., 2020; Delage et al., 2021). Concurrently, they display similarities with the early developmental period, a stage when microglia actively participate in the assembly and refinement of neural circuits (Schafer et al., 2012; Hammond et al., 2019). Parallel to an increase in the diversity of aging-related molecular signatures, aged microglia are morphologically altered when observed using light microscopy and tend to adopt more of an amoeboid-shaped phenotype. Alternatively, increased density of rod-shaped microglia (Bachstetter et al., 2017) or dystrophic microglia with reduced, gnarly processes have been described (Streit et al., 2004; Damani et al., 2011). These alterations may be of direct relevance to the functional irregularities reported in aged microglia. In this line of idea, transcriptomic investigation in human microglia reveals lower expression of CX3CR1 in aging (Galatro et al., 2017). Similar investigation in stressed microglia showed clustering of microglial population for genes related to inflammation and cytokine secretion (Lehmann et al., 2018), with the main ones illustrated in Figure 1. Furthermore, the overall microglial response to stimuli seems to be poorly managed with aging, suffering delays as well as prolonged duration, with potential detrimental consequences for the integrity of the aged CNS (Damani et al., 2011; Hefendehl et al., 2014; Jiang et al., 2020). Long-term sustenance of non-homeostatic microglial states in combination with morphological changes and loss of motility interfering with their optimal area surveillance (Damani et al., 2011; Hefendehl et al., 2014) may be contributing to an excessive accumulation of debris. Underlying mechanisms of the excessive debris accumulation may include increased lysosomal pH and compromised debris clearance notably through phagocytosis. This might be caused by their inability to protect and maintain the physiological function of neurons, synapses, myelin, etc. via trophic factors (Solé-Domènech et al., 2018; Pluvinage et al., 2019). Of note, the concept of senescence, a state when a cell enters division cycle arrest but preserves its metabolic activity and possesses a modified senescence-associated secretory phenotype (Coppé et al., 2010), has recently been a trending topic in the microglial field. Current evidence demonstrated that microglia can preserve their proliferative capacity even in aging as acutely extracted microglial cells from aged murine brains seem to lack up-regulation of senescence-associated markers, e.g., beta-galactosidase, as compared to long-term cultured microglia (Stojiljkovic et al., 2019; Hu et al., 2021). Previously, dystrophic morphological traits have been suggested for this phenotype (Streit et al., 2004). It is yet to be established whether senescent microglia are an independent microglial subtype of aged microglia or a state within the course of microglial aging.

Aged microglia possess elevated levels of mature lysosomal structures, undegradable lipofuscin granules and lipid bodies that are also larger in size (Sierra et al., 2007; Safaiyan et al., 2016; Marschallinger et al., 2020). Moreover, as was shown in a mouse model, aged microglia display reduced expression of the essential autophagy gene, *Atg7* (Berglund et al., 2020), suggesting a limited intracellular maintenance. Metabolic failure occurs alongside impairments in the mitochondrial electron transport chain (ETC) during aging, identified by an altered/abnormal state of mitochondrial cristae ultrastructure, a marker of oxidative stress (Minhas et al., 2021). Upon exposure to challenges, microglia can undergo a switch from the efficient homeostatic oxidative metabolism (OXPHOS) to a faster ATP production via aerobic glycolysis, to sustain their elevated activity and increased coping strategies (Lauro and Limatola, 2020). In aging, this mechanism may become maladaptive, leading to the formation and accumulation of glycogen (a glucose storage), accompanied by a decreased mitochondrial respiration via the prostaglandin E2 (PGE2) and its receptor (EP2) pathway, as recently shown in mice (Minhas et al., 2021). Microglial proteomic analysis further suggested that this aging-related metabolic shift may relate to their utilization of alternate substrates, such as fatty acid, thus contributing to increased cholesterol trafficking with aging, which may result in neuronal dysfunction and cognitive decline (Flowers et al., 2017). Higher levels of neuronal cholesterol are considered detrimental, as myelinogenesis is slowed in aging, coincident with cytotoxic accumulation of cholesterol and cognitive decline (Kadish et al., 2009). On top of that, increase of intracellular iron, possibly related to an accumulation of lipofuscin, which is able to bind metals (Höhn et al., 2010), may be a contributing factor to aging-related oxidative imbalance and OXPHOS failure. As such, iron overload is considered one of the hallmarks of aged or senescent microglia (Angelova and Brown, 2019).

Several intracellular microglial properties were shown to be affected by aging (Cho et al., 2019). In agreement with observations made in other cell types, aged microglia show several signs of cellular aging such as epigenetic changes via the hypomethylation of IL-β associated with elevated Il-β transcript in microglia (Cho et al., 2015), which are described in Figure 1, along with an impaired DNA repair capability, altered transcription machinery and diminished ability of chromatin remodeling, as revealed by proteomic analysis of aged primary mouse microglia (Figure 1; Raj et al., 2014; Flowers et al., 2017; Costa et al., 2021). This inadequate microglial remodeling may underlie their impaired ability to dynamically change states according to the needs of the CNS during aging (Flowers et al., 2017). It is important to highlight that the telomere length is chemically affected by oxidative stress, notably via hydrogen peroxidase (H_2_O_2_), a reactive substrate that can cause prominent DNA damage (Oikawa and Kawanishi, 1999). Together, these cellular changes should be prevented or rescued to promote healthy aging (Depp et al., 2007; Wolkowitz et al., 2011), especially among key brain regions that are impacted by stress and aging.

## Brain Hippocampus and Prefrontal Cortex: Key Regions Impacted by Stress and Aging

### The Hippocampus

Hippocampal involvement in memory and learning has been widely studied across mouse, rat, non-human primate and human (Jarrard, 1993; Banta Lavenex and Lavenex, 2009; Voss et al., 2017; Zalucki et al., 2018). While still debated in human (Rodríguez-Iglesias et al., 2019), neurons in animal models are continuously produced throughout the lifespan among the olfactory bulb (Breton-Provencher et al., 2009) and hippocampus (Toda et al., 2019), while microglia also maintain their population by self-renewal (Tay et al., 2017). Several studies indicate that microglia may participate in the hippocampal-dependent learning and memory processes (Jarrard, 1993; Banta Lavenex and Lavenex, 2009; Parkhurst et al., 2013; Torres et al., 2016; Voss et al., 2017; Zalucki et al., 2018). For instance, microglial ablation via the CX3CR1-diphtheria toxin receptor reduces performance in object recognition test in rats (De Luca et al., 2020). The hippocampal CA1 makes projection to other brain regions also affected by stress, including the PFC (Rosene and Van Hoesen, 1977). This association seems to place the hippocampus as the gateway network particularly activated during learning tasks, as shown using magnetic resonance imaging (MRI) in human (Doyère et al., 1993; McEwen et al., 2016). It was also shown that stress resulting from sleep deprivation decreases hippocampal neurogenesis and induces depressive behavior (Murata et al., 2018; Han et al., 2019a). This contrasts with the ability of newborn neurons to contribute to stress resilience by inhibiting the hippocampal dentate gyrus mature granule cells activity as measured using electrophysiology (Anacker et al., 2018). The hippocampal functions are highly sensitive to accelerated aging, showing reduced cholinergic inputs and diminished neuronal activity rhythm in a mouse model of AD pathology (Rubio et al., 2012). Microglia regulate hippocampal neurogenesis in development (Pérez-Rodríguez et al., 2021) and throughout life (Reshef et al., 2017; Szepesi et al., 2018; Augusto-Oliveira et al., 2019; Bordeleau et al., 2019; Tan et al., 2020), in both health and neurodegeneration (De Lucia et al., 2016), via an IL-4 driven brain-derived neurotrophic factor (BDNF)-dependent mechanism (Zhang et al., 2021). Microglial influence on cognition could result from their roles in neurogenesis and at synapses. Indeed, these roles discussed in the section on fractalkine signaling can be performed notably via the secretion of inflammatory mediators and neurotrophic factors (Parkhurst et al., 2013; Sierra et al., 2014). Furthermore, microglia contribute to the control of synaptic activity and plasticity, constituting a quad-partite synapse alongside astrocytes, notably in the hippocampus (Bennett, 2007; Tremblay et al., 2011, 2014; Béchade et al., 2013; Schafer et al., 2013; Sierra and Tremblay, 2014).

### The Prefrontal Cortex

The PFC is a key area affected by stress, with major impact on the behavior across species (Arnsten and Goldman-Rakic, 1998; Cerqueira et al., 2007; Holmes and Wellman, 2009; Myers-Schulz and Koenigs, 2012; Arnsten et al., 2015; McKlveen et al., 2015; Page et al., 2019). The stress response involves complex changes in neuron-microglia-astrocyte relationships, notably denoted by increased microglial phagocytosis of dendritic spines, as well as reduced astrocytic coverage areas in the PFC of rats exposed to chronic stress (Woodburn et al., 2021). This is in line with data showing an altered microglial morphology in rats after chronic stress, with females showing a greater proportion of primed microglia compared to males (Bollinger et al., 2016). Studies performed in aging are nevertheless lacking. Furthermore, PFC-dependent functions such as spatial working memory are affected by chronic stress in aged humans with MDD (Beats et al., 1996; Nebes et al., 2000) and in aged rats, and correlate with increase in staining intensity of ionized calcium binding adapter molecule 1 (Hinwood et al., 2012), a microglia/infiltrating macrophage marker (Imai et al., 1996). In a rat model of working memory deficiency, performance during the task was improved by administering minocycline (Hinwood et al., 2012), which normalizes microglial inflammatory and phagocytic functions (Mattei et al., 2014, 2017), potentially rescuing the homeostatic microglia-neuron crosstalk (Miao et al., 2018). As shown in the hippocampus during aging and in the PFC upon chronic stress, time is of the essence as studies show deleterious effects over time (Hanamsagar and Bilbo, 2017; Angelova and Brown, 2019), notably due to chronic inflammation acting in a negative feedback manner (Comer et al., 2020).

## Microglial Involvement in Stress and Cognitive Decline Assessed in Human

**FIGURE 2:**
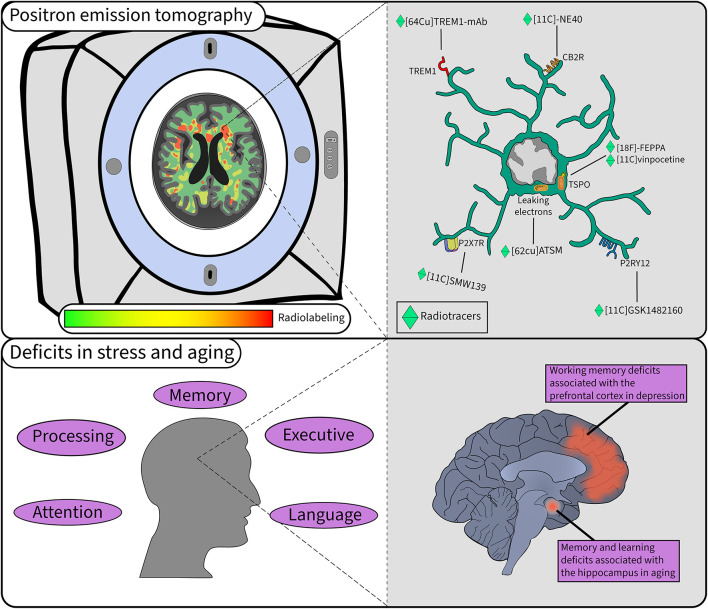
Microglial study using PET in stress and aging. PET is a widely used technique when investigating microglial activity in pathology. In the top left, microglial labeling of radiotracers is quantified to provide insight into their function in the human brain in a non-invasive manner. In the top right a myriad of tracers provide different information on the function of microglia in the brain. The bottom left panel highlights some of the cognitive deficits observed in aging that can be accelerated with chronic stress. The bottom right panel illustrates the key regions and deficits associated with stress and aging.

In human, the cognitive abilities of aged or chronically stressed subjects can be assessed on multiple levels. One of them is cumulative knowledge (crystallized intelligence) including general knowledge with vocabulary and historical knowledge, the other being the cognitive processing (fluid intelligence) of information in order to complete a task (Paterniti et al., 2002; Wilson et al., 2013; Murman, 2015). Extensive work from Harada et al. (2013) highlighted deficits in processing speed when performing tasks, attention, memory and language with aging. These deficits were highly correlated with gray and white matter volume reductions revealed by MRI (Harada et al., 2013). A reduced executive control in aging can also be detected using the Wisconsin Card Sorting Task as an impairment often associated with function and volume diminution of the PFC and other frontal areas (Ballesteros et al., 2013). Patients with MDD experience deficits in attention, learning, as well as in a short-term and working memory (Lam et al., 2014). This is accompanied by evidence showing changes in white matter microstructures in adults with MDD including the cingulum and the hippocampus (van Velzen et al., 2020). There is then a clear link between cognitive deficit, stress and anatomical changes in the brain (Lam et al., 2014; van Velzen et al., 2020), but one might ask how this process occurs at the cellular level. Microglial activity can be measured with PET using the coupling of a radiotracer, most commonly [^11^C]PK11195 (Best et al., 2019) or [^11^C]PBR28 (Nettis et al., 2020), and its receptor Translocator Protein 18 kDa (TSPO) (Figure 2). TSPO has diverse cellular functions, and increases in its expression can signify impaired mitochondrial metabolism, increased oxidative stress, phagocytosis, and inflammation (Shoshan-Barmatz et al., 2019), particularly in glial cells (Pannell et al., 2020). Using this tracer, it was shown that elevated microglial PET activity parallels a decline in cognition with aging evaluated by cognitive processing (Parbo et al., 2017). Hence, the aging population is likely to suffer cognitive symptoms which can be triggered and/or escalated by chronic psychological stress and other environmental factors (Gonçalves de Andrade et al., 2021; Rizzo and Paolisso, 2021).

Measuring TSPO to infer glial activity is informative in various neurological diseases including AD, but also mild cognitive impairment (Hannestad et al., 2013; Holmes et al., 2018; Best et al., 2019; Kreisl et al., 2020; Meyer et al., 2020), and cognitive aging (Gulyás et al., 2011; Suridjan et al., 2014). Most studies have reported increased TSPO expression in patients with MDD compared to healthy controls, and higher levels were measured the longer MDD remained untreated (Meyer et al., 2020). PET studies on aging have reported mixed results (Repalli, 2014), with one using [^18^F]-FEPPA showing no increase in TSPO levels across the lifespan in healthy individuals (Suridjan et al., 2014), while another using [^11^C]vinpocetine found that TSPO expression increased linearly with age (Gulyás et al., 2011). A study by Ottoy et al. (2019) found that reduced ^18^F-fluorodeoxyglucose (^18^FDG) PET uptake, indicative of brain hypometabolism, was strongly associated with short-term cognitive decline and predicted a short-term transition to AD in mildly cognitively-impaired patients. Inconsistencies between studies may be in part due to limitations with TSPO radiotracers such as low signal-to-noise ratio or partial blood-brain barrier permeability (Kreisl et al., 2020). Moreover, limitations exist with the TSPO target itself, as it is not microglia-specific, being also expressed by astrocytes, endothelial cells and other myeloid cell types which can also be at play upon stress and in aging (Janssen et al., 2018; Carrier et al., 2020). Certain alternatives to TSPO, like the TREM and purinergic family of receptors, which are summarized in Figure 2, have gained interest in recent years to assess microglial function and their interactions with neurons in human (Janssen et al., 2018; Narayanaswami et al., 2018). Other than measuring mitochondrial activity, mitochondrial dysfunction can be imaged with ^62^Cu-ATSM [Cu-diacetyl-bis(*N*^4^-methylthiosemicarbzone)], which tags excessive electron buildup caused by leakage from malfunctioning ETC, suggesting an accumulation of reactive oxygen species (ROS) (Ikawa et al., 2020). ROS might be of particular interest as their cerebral levels are known to increase with stress and aging (Seo et al., 2012; Narayanaswami et al., 2018), limiting microglial ability to interact properly with neurons (Qin et al., 2002; Hur et al., 2010; Spencer et al., 2016; Munoz et al., 2017). Other investigations have developed and tested radiotracers targeting the myeloid cell marker CSF1R, able to track microglia in health and disease (Horti et al., 2019). However, there is more work required to expand the PET imaging radiotracer panel to understand human microglia and neuron crosstalk.

## Discussion

Chronic stress was proposed to accelerate the process of cellular aging and promote sensitization or *priming* of microglia toward external stressors (Franklin et al., 2018; Rentscher et al., 2019). This over-sensitization by psychological stress is related to immunosenescence, i.e., the accumulation of oxidative stress causing deficit in responses revolving around the immune system (Gingrich, 2005; Deleidi et al., 2015) and preventing appropriate communication of microglia with the local environment, reported as hostile (Cornejo and von Bernhardi, 2016). The microglial sensome was identified as a key defective player during cognitive aging in the hippocampus and PFC (Kerr et al., 1991; Miller and O’Callaghan, 2005; Bauer, 2008; Bloss et al., 2010; Tay et al., 2018, 2019; Zannas et al., 2018). Figure 1 shows how psychological stress can alter the communication between microglia and neurons via inflammation leading to cellular aging of both neurons and microglia through multiple processes. In particular, aged microglia show morphological signs of dystrophy preventing their homeostatic function and often associated with neurodegenerative disease (Angelova and Brown, 2019; Savage et al., 2019; Swanson et al., 2020; Shahidehpour et al., 2021). Overall, microglia in pathological aging recruit different pathways compared to younger ages, with a predominant presence of inflammatory ones including TREM2, TAM and complement signaling (Flowers et al., 2017; Tay et al., 2017; Ulland et al., 2017; Pan et al., 2020; Keane et al., 2021). Microglial TREM2 has been particularly linked to aging, with 24 month old TREM2 knockout mice showing fewer and less phagocytic microglia based on a lower expression of CD68 in brain samples (Linnartz-Gerlach et al., 2019). Future studies are needed to investigate this change in TREM2 expression in humans experiencing chronic stress along the aging trajectory by PET imaging combined with a psychological cognitive assessment. Furthermore, the dynamics of purinergic receptors also require further investigation to determine if A1R is possibly affected in stress or aging, particularly if a disbalance in P2RY12/A1R may be at play.

There are also genetic predispositions known to accelerate aging by altering the microglial responses to stress. Genetic ablation of the protease cathepsin B (CatB) reduces oxidative stress in mice, thus improving cognitive performance with aging (Ni et al., 2019). Other types of stressors such as obesity-induced oxidative stress can accelerate the adverse effects of aging (Tarantini et al., 2018), resulting in increase of oxidative radicals in microglia which in turn contribute to the accelerating aging process (Raj et al., 2015). It is important to note that even if the role of microglia in oxidative stress is substantial, neurons in culture without glial cells can also show oxidative stress, meaning that glial cells are not solely responsible for brain cellular stress (Sompol et al., 2008; Hui et al., 2016). Indeed, neuronal inflammation is a major contributor to the Werner syndrome mouse model of premature aging, a neurological disorder associated with neuronal oxidative stress and microglial morphological changes (Hui et al., 2018). The overall outcome of impaired microglial aging may lead to impairments of synaptic plasticity, neurogenesis, accompanied by a loss of functions of other glial cells such as oligodendrocytes (Baror et al., 2019) and pathological neovascularization (Jiang et al., 2020). Oligodendrocytes are responsible for the CNS myelination and alteration of these cells contributes to memory deficits in aged mice (Wang et al., 2020). As these processes may play a role in aging-associated cognitive decline, targeting microglia presents an innovative direction of the therapeutic research. Potential therapies that could limit or rescue microglial aging, normalizing their key roles in learning and memory and the adaptation to environmental challenges, most notably include aerobic exercise and stress management in humans, also shown using wheel-running and outdoor living in mice (Tosato et al., 2007; Niraula et al., 2017; Yegorov et al., 2020). Dietary interventions like caloric restriction, in order to limit cellular cytotoxicity and promote anti-inflammatory activities, have also shown a great potential to promote healthy aging (Cheng et al., 2010). For instance, by inhibiting the mTOR pathway (López-Lluch and Navas, 2016) and thus potentially normalizing TREM2 signaling (Zhou et al., 2018), diet could lead to more efficient microglia when looking at the phagocytic ability and response ability to stress. Dietary intervention using ketogenic food made from soybean oil and cocoa butter is also beneficial in mice subjected to chronic stress (Guan et al., 2020; Morris et al., 2020). Similar dietary intervention is also demonstrating high potential to reducing mild cognitive impairment encountered in aging (Fortier et al., 2019, 2021). While we have presented preventive options, other curative therapies have shown interest. For instance, targeting survival signaling by blocking CSF1R was found to reduce Alzheimer’s-like pathology in mice (Olmos-Alonso et al., 2016). The monitoring of stress and aging in patients using PET imaging and psychological testing for cognitive decline seems like an interesting strategy to promote healthy aging, even more when paired with diet and healthy living.

## Conclusion

This work aimed to focus on potential therapeutic targets and highlight preventive therapies that may be beneficial to limit the development of MDD and accelerated aging, as they are often less covered in the literature because of their long-term reward on the patient health. Using the communication pathways altered in stress and aging summarized in Figure 1, we can thus orient the development of new radiotracers and investigate pre-existing ones as shown in Figure 2, which would allow to monitor microglia-neuron communication in MDD. Some major regions of the brain are altered in stress and aging such as the hippocampus and the PFC, although other regions are also targeted like the insular lobe. Finally, this work proposes the long-term goal of preventing and identifying novel biomarkers for MDD and accelerated cognitive aging using translational research.

## Author Contributions

MC was the principal manager of the review, wrote the discussion, section on human brain regions, and microglial-neuron communication, took care of the overall revision and formatting of the manuscript, and created the figures included in the manuscript. EŠ oversaw writing of the microglial metabolic changes in stress and aging section. CM oversaw redaction of the PET imaging section. M-KS-P was responsible for writing the introduction. M-ÈT oversaw the conceptual framework of the review while contributing significantly to the organization, design, and revision of the manuscript. All authors contributed to the article and approved the submitted version.

## Conflict of Interest

The authors declare that the research was conducted in the absence of any commercial or financial relationships that could be construed as a potential conflict of interest.

## Publisher’s Note

All claims expressed in this article are solely those of the authors and do not necessarily represent those of their affiliated organizations, or those of the publisher, the editors and the reviewers. Any product that may be evaluated in this article, or claim that may be made by its manufacturer, is not guaranteed or endorsed by the publisher.
